# Snapin Specifically Up-Regulates Ca_v_1.3 Ca^2+^ Channel Variant with a Long Carboxyl Terminus

**DOI:** 10.3390/ijms222011268

**Published:** 2021-10-19

**Authors:** Sua Jeong, Jeong-Seop Rhee, Jung-Ha Lee

**Affiliations:** 1 Department of Life Science, Sogang University, 35 Baekbeom-ro, Mapo-gu, Seoul 04107, Korea; suajeong@sogang.ac.kr; 2 Synaptic Physiology Group, Department of Molecular Neurobiology, Max Planck Institute for Experimental Medicine, Hermann-Rein-Str. 3, 37075 Göttingen, Germany; rhee@em.mpg.de

**Keywords:** snapin, Ca_v_1.3 L-type Ca^2+^ channel, two-electrode voltage clamping, whole-cell patch clamping, immunostaining, ON-gating current

## Abstract

Ca^2+^ entry through Ca_v_1.3 Ca^2+^ channels plays essential roles in diverse physiological events. We employed yeast-two-hybrid (Y2H) assays to mine novel proteins interacting with Ca_v_1.3 and found Snapin2, a synaptic protein, as a partner interacting with the long carboxyl terminus (CT_L_) of rat Ca_v_1.3_L_ variant. Co-expression of Snapin with Ca_v_1.3_L_/Ca_v_β_3_/α_2_δ_2_ subunits increased the peak current density or amplitude by about 2-fold in HEK-293 cells and *Xenopus* oocytes, without affecting voltage-dependent gating properties and calcium-dependent inactivation. However, the Snapin up-regulation effect was not found for rat Ca_v_1.3_S_ containing a short CT (CT_S_) in which a Snapin interaction site in the CT_L_ was deficient. Luminometry and electrophysiology studies uncovered that Snapin co-expression did not alter the membrane expression of HA tagged Ca_v_1.3_L_ but increased the slope of tail current amplitudes plotted against ON-gating currents, indicating that Snapin increases the opening probability of Ca_v_1.3_L_. Taken together, our results strongly suggest that Snapin directly interacts with the CT_L_ of Ca_v_1.3_L_, leading to up-regulation of Ca_v_1.3_L_ channel activity via facilitating channel opening probability.

## 1. Introduction

External Ca^2+^ entry through voltage-activated Ca^2+^ channels (VACCs) acts as Ca^2+^ signals to mediate numerous physiological functions. VACCs are composed of a pore-forming α_1_ subunit for Ca^2+^ entry and auxiliary subunits. Molecular cloning uncovered 10 Ca^2+^ channel α_1_ subunits, which determine primary electrophysiological and pharmacological characteristics of VACCs. In accordance with their electrophysiological and pharmacological properties as well as sequence similarity, Ca_v_1.1-1.4 are classified as L-type Ca^2+^ channels, Ca_v_2.1-2.3 as non-L-type Ca^2+^ channels, and Ca_v_3.1-3.3 as T-type Ca^2+^ channels [1].

The L-type channels play distinctive roles with different tissue distribution. Ca_v_1.1 acts as a voltage sensor in excitation-contraction coupling in skeletal muscle. Ca^2+^ influx through Ca_v_1.2 triggers contraction of cardiac and smooth muscle. Although Ca_v_1.2 and Ca_v_1.3 are commonly expressed in various tissues, their differential roles have been reported. For instance, Ca_v_1.2 mediates cardiac myocyte contraction in the heart, whereas Ca_v_1.3 contributes to pacemaker activity in the sinoatrial (SA) node [2,3]. In addition, Ca_v_1.4 is expressed predominately in the retina and was originally identified by its mutation being associated with congenital night blindness [4]. 

Studies of Ca_v_1.3 knock-out mice showed malfunction and degeneration of afferent auditory nerve fibers and hair cells, cardiac bradycardia arising from decreased pacemaker activity, hippocampal learning and memory impairment via reduced survival of new-born neurons, and insulin secretion reduction from pancreatic β cells [2,3,5,6]. More recently, missense mutations in Ca_v_1.3 have been implicated in primary aldosteronism [7] and neuronal diseases, such as autism spectrum disorders and seizures [8,9]. Because of relatively low activation threshold, certain Ca_v_1.3 variants have been suggested as being low voltage-activated L-type Ca^2+^ channels and crucially involved in hormone secretion and certain neuronal excitability in response to low potential [10,11,12,13]. Indeed, the low-voltage activation of Ca_v_1.3 channels in post-synaptic neurons and adrenal chromaffin cells was found to trigger consecutive spikes and post-bust after-hyperpolarization via functional association with Ca^2+^-activated K^+^ channels [12,14,15]. Furthermore, Ca_v_1.3 channels were reported to influence dendritic spine morphology and synaptic plasticity [8,16]. 

Snapin was initially found to associate with SNAP-25, a component of trans-SNARE complex in neuronal tissues to promote and stabilize SNARE complex assembly, leading to facilitation of synaptic transmission processes [17,18]. In spite of a challenging report in which the low expression and minor role of Snapin in neurons was addressed [19], Snapin deletion studies were shown to impair hormone secretion, synaptic vesicle fusion, and the homeostatic modulation of presynaptic vesicle release [20,21,22], supporting the facilitating effect of Snapin on synaptic transmission and hormone secretion. In non-neuronal cells, Snapin was found to be ubiquitously expressed and detected to interact with SNAP-23 [23]. As a component of BLOC1 (biogenesis of lysosome-related organelles) complex, Snapin was reported to contribute to the biogenesis of lysosome-related organelles and the intracellular transport of endocytosed materials for degradation [24,25]. Snapin, which mediates endo-lysosomal transport, was also involved in the recycling of synaptic vesicles in neuronal tissues [26]. Furthermore, the potential roles of Snapin have been greatly expanded with findings of its diverse interaction partners, including ryanodine receptor [27], water channels [28], TRPV1 [29], and Ca_v_1.3 Ca^2+^ channel α_1_ subunit [30]. In the latter report, Snapin1 co-expression in HEK-293 cells stably expressing Ca_v_1.3 α_1_ plus auxiliary subunits was shown to strongly reduce the peak current amplitude of Ca_v_1.3 channels. Similarly, Snapin1 overexpression in HL-1 cells was shown to profoundly reduce endogenous Ca_v_1.3 channel activity and protein. The Snapin-mediated ubiquitination of Ca_v_1.3 was addressed to be involved in the underlying mechanism for the reduction of Ca_v_1.3.

Using yeast-two-hybrid (Y2H) screenings, in the present study, we attempted to mine molecular partners interacting with the long carboxyl terminus (CT_L_) of rat Ca_v_1.3_L_ and isolate Snapin2, the broad roles of which were reviewed in the above paragraph. In contrast with a previous report [30], our reconstitution experiments of Ca_v_1.3_L_ and Snapin in HEK-293 cells and *Xenopus* oocytes showed that Snapin co-expression similarly increased the channel activity of Ca_v_1.3_L_ in the two systems, without changing voltage-dependent gating properties. Combined studies using immunocytochemistry and electrophysiology uncovered that the Snapin up-regulation of Ca_v_1.3_L_ current arises from increased opening probability without changing surface expression.

## 2. Results

### 2.1. Snapin2 Is an Interaction Partner of Rat Ca_v_1.3_L_

**Figure 1:**
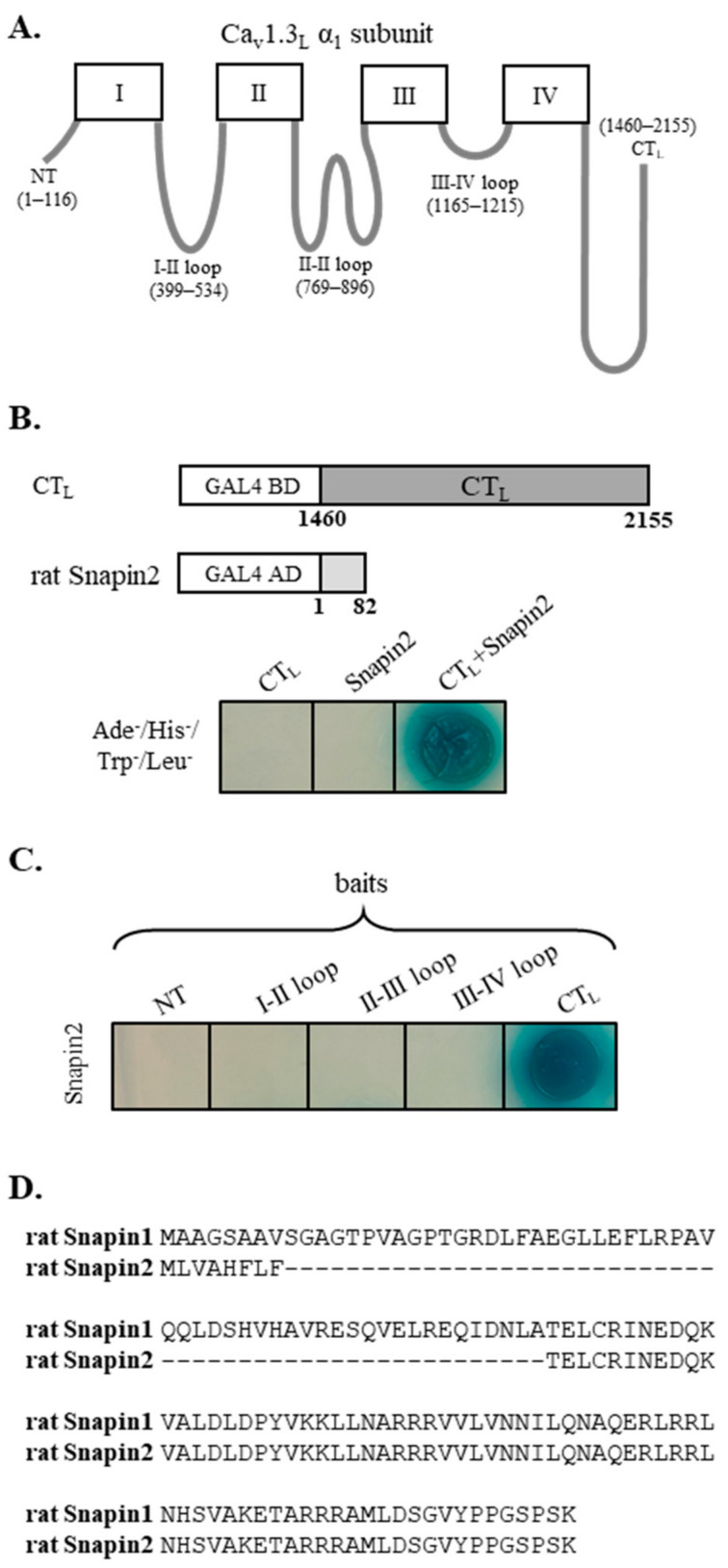
Isolation of rat Snapin2 as an interaction partner with the CT_L_ of rat Ca_v_1.3 by yeast-two-hybrid (Y2H) screenings. (**A**) Schematic diagram of rat Ca_v_1.3_L_ α_1_ subunit. The amino acid numbers for the intracellular structures of rat Ca_v_1.3_L_ α_1_ subunit, including NT (amino terminus), I–II, II–III, III–IV, and CT_L_ are designated in parentheses. (**B**) Y2H data representing an interaction between the CT_L_ and rat Snapin2. A positive clone containing rat Snapin2 construct was isolated from Y2H screenings for which the CT_L_ and a rat brain library were used as bait and prey, respectively. Co-transformation of the CT_L_ and rat Snapin2 produced a blue colony in a high-stringent condition (−Ade, −His, −Leu, and −Trp) with X-gal. (**C**) Y2H results illustrating that rat Snapin2 interacted with only the CT_L_ but not with the other intracellular structures. (**D**) Amino acid sequence alignment between rat Snapin1 and Snapin2.

We searched for novel molecular partners interacting with Ca_v_1.3 Ca^2+^ channel, employing Y2H screening, for which the long carboxyl terminus (designated as CT_L_, Figure 1A) of rat Ca_v_1.3_L_ was used as bait, and a rat brain cDNA library was used as prey. A positive blue clone grown in a high-stringent condition (Ade^−^, Leu^−^, His^−^, Trp^−^) was isolated. The vector insert was sequenced and analyzed by BLAST, which identified that the DNA sequence contained the whole open-reading frame (82 amino acids) of rat Snapin2 (Genbank no. NM_001025648). Sequence comparison (Figure 1D) showed that rat Snapin2 is a shorter variant than rat Snapin1 (136 amino acids). Notably, rat Snapin2 amino acid sequence approximately corresponds to the post half sequence (amino acid number 63 to 136) of rat Snapin1 When the CT_L_ and Snapin2 were co-transformed, positive blue colonies were formed in a high-stringent condition in the presence of X-gal, confirming their interaction (Figure 1B). Additional Y2H assays were conducted to examine whether Snapin2 can interact with the other cytoplasmic structures of Ca_v_1.3_L_, including the amino-terminus (NT) and cytoplasmic loops connecting domains I, II, III, and IV (Figure 1A). It was determined that Snapin2 interacted with only the CT_L_ of Ca_v_1.3_L_ but did not with the other cytoplasmic tail and loops (Figure 1C).

### 2.2. Up-Regulation of Ca_v_1.3_L_ Channel Activity by Snapin2 in HEK-293 Cells 

**Figure 2:**
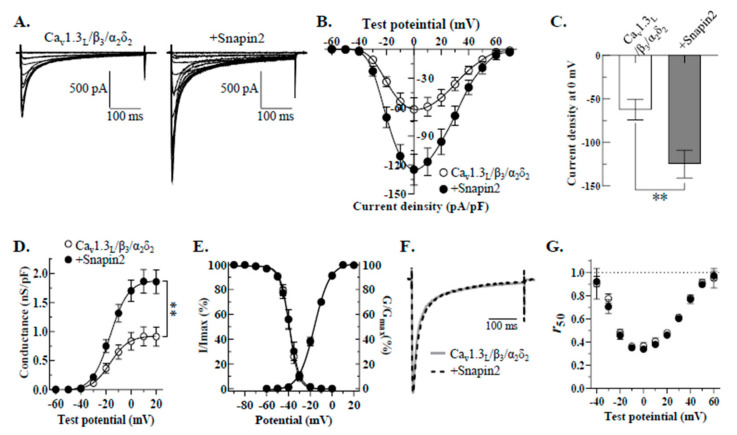
Up-regulation of Ca_v_1.3_L_ channel activity by Snapin2 in HEK-293 cells. (**A**) Representative Ca^2+^ current traces through Ca_v_1.3_L_ channels co-expressed without (left) or with Snapin2 (right). Ca_v_1.3_L_, β_3_, and α_2_δ_2_ cDNA constructs were transfected into HEK-293 cells (left) and co-transfected with rat Snapin2 cDNA construct in an equal molar ratio (right). In 10 mM Ca^2+^ solution, Ca^2+^ current traces through Ca_v_1.3_L_ channels were evoked by application of an *I-V* protocol consisting of 400-ms depolarizing step pulses from −60 mV to +70 mV by 10 mV increments from a holding potential of −80 mV. (**B**,**C**) *I-V* relationships of Ca_v_1.3_L_ channels co-expressed without or with Snapin2. Current density values (pA/pF) obtained by dividing the peak current amplitude values of Ca_v_1.3 Ca^2+^ currents evoked at various test potentials by cell capacitance were averaged and then plotted against test potentials (n = 13–15). (**C**) The maximal peak current density values of Ca_v_1.3_L_ channels co-expressed without or with Snapin2 measured at 0 mV test potential are represented as bar graphs (−62.3 ± 11.9 vs. −124.8 ± 15.9 pA/pF; n = 13–15; Student’s unpaired *t*-test, *p <* 0.01, **). (**D**) Snapin2 effect on the chord conductance of Ca_v_1.3_L_ channels. The chord conductance values of Ca_v_1.3_L_ channels without or with Snapin2 were normalized to the cell capacitance, and average values were plotted against test potentials. Snapin2 co-expression increased the maximal conductance of Ca_v_1.3_L_ by roughly 1.9-fold at 10 mV (0.9 ± 0.2 vs. 1.9 ± 0.2 nS/pF; n = 13–15; Student’s unpaired *t*-test, *p <* 0.01, **). (**E**) Snapin2 effect on voltage-dependent channel activation and channel availability of Ca_v_1.3_L_ channels. The chord conductance values of Ca_v_1.3_L_ channels without or with Snapin2 were normalized and superimposed (n = 13–15). Channel availability of Ca_v_1.3_L_ channels was evaluated by a two-step pulse protocol consisting of 10-s pre-conditioning pulses ranging from −90 mV to 0 mV by an increment of 10 mV, followed by a step pulse to 0 mV. Currents measured at 0 mV test potential were normalized to the current amplitude after a preconditioning potential of −90 mV and plotted against pre-pulse potentials. The average percentage data were fit to the Boltzmann equation (n = 10–12). (**F**,**G**) Snapin2 effect on CDI of Ca_v_1.3_L_ channels. (**F**) Ca_v_1.3_L_ Ca^2+^ current traces through Ca_v_1.3_L_ channels without or with Snapin2 at 0 mV test potential were normalized and superimposed, exhibiting little difference in CDI. (**G**) Snapin effect on *r*_50_. The *r*_50_ values (the fractions of Ca^2+^ peak currents remaining after 50 ms; n = 7–8) of Ca_v_1.3_L_ channels without or with Snapin2 were plotted against test potentials. The *r*_50_ values were not altered by Snapin2. No differences in the *r*_350_ values were detected (data not shown).

To characterize the effects of Snapin2 on Ca_v_1.3_L_, Snapin2 was co-expressed with Ca_v_1.3_L_/Ca_v_β_3_/α_2_δ_2_ by transfecting the cDNA constructs into HEK-293 cells. We recorded Ca_v_1.3_L_ channel currents from HEK-293 cells using whole-cell patch clamping configuration from 48 h after transfection. Upon a series of 400-ms depolarizing step pulses ranging from −60 mV to +70 mV from a holding potential of −80 mV, robust Ca^2+^ current traces were evoked from cells expressing Ca_v_1.3_L_/Ca_v_β_3_/α_2_δ_2_ (Figure 2). The evoked Ca_v_1.3_L_ currents were immediately activated upon serial step pulses, followed by rapidly decaying kinetics during the given period of test potentials, representing typical calcium-dependent inactivation (CDI) properties (Figure 2A). The current-voltage relationships (Figure 2B) obtained by plotting average current density values versus test potentials showed that the threshold potential was about −40 mV, the potential inducing maximal peak current was 0 mV, and the reversal potential was around +70 mV. These biophysical properties are quite similar to those reported previously [31]. Notably, the Ca^2+^ current densities over most of the test potentials were greatly facilitated by co-expression of Snapin2 with Ca_v_1.3_L_ and auxiliary subunits (Figure 2A,B). The maximal peak current density values at 0 mV test potential were −62.3 ± 11.9 pA/pF for Ca_v_1.3_L_/Ca_v_β_3_/α_2_δ_2_ (control) and −124.8 ± 15.9 pA/pF for co-expression of Snapin2, indicating that the Snapin2 approximately doubled the Ca_v_1.3_L_ channel activity (Figure 2C). Similarly, the maximal chord conductance of Ca_v_1.3_L_ channels was two-fold increased by Snapin2 (0.92 ± 0.16 vs. 1.86 ± 0.20 nS/pF, Figure 2D). Besides the up-regulation in current density, we found that Snapin2 co-expression induced no significant alterations in other biophysical properties, including voltage-dependent gating properties and CDI (Figure 2E–G; Table 1).

### 2.3. The Snapin2 Up-Regulation Effect on Ca_v_1.3_L_ Channel Was Reproduced in Xenopus Oocytes

**Figure 3:**
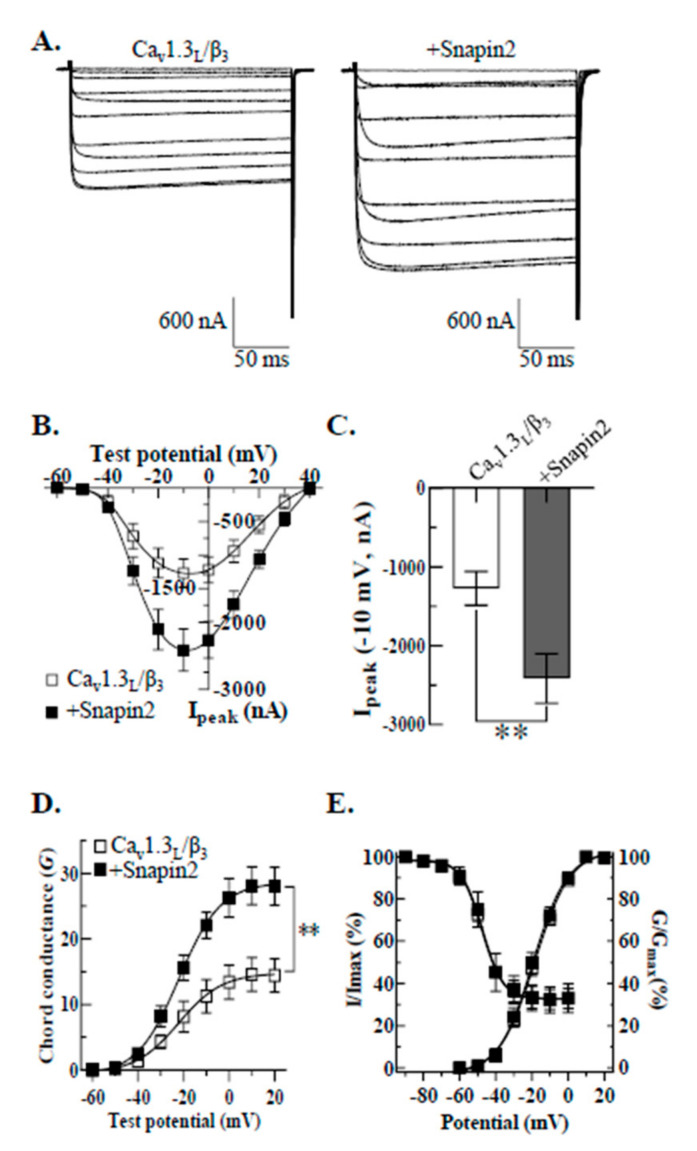
Snapin2 co-expression up-regulated Ca_v_1.3_L_ channel activity in *Xenopus* oocytes. (**A**) Representative current traces of Ca_v_1.3_L_ and Ca_v_β_3_ co-expressed without (left) or with Snapin2 (right) recorded in 10 mM Ba^2+^ solution. Current traces were evoked by an *I-V* protocol consisting of 200-ms depolarizing step pulses from −60 mV to +40 mV from a holding potential of −80 mV. (**B**,**C**), *I-V* relationships of Ca_v_1.3_L_ channels without or with Snapin2. (**B**) Average peak current amplitude values of Ca_v_1.3_L_ without (□) or with Snapin2 (■) were plotted against test potentials (n = 20–23). (**C**) The average peak current amplitude values of Ca_v_1.3_L_ channels at −10 mV test potential are represented as bar graphs (−1271.1 ± 214.1 vs. −2414.5 ± 314.6 nA; n = 20–23; Student’s unpaired *t*-test, *p <* 0.01, **). (**D**) Chord conductance of Ca_v_1.3_L_ channels co-expressed without (□) or with Snapin2 (■). Snapin2 co-expression enhanced about 1.9-fold the maximum chord conductance values (14.6 ± 2.6 vs. 28.1 ± 2.9 nS; n = 20; Student’s unpaired *t*-test, *p <* 0.01, **). (**E**) Snapin2 effect on voltage-dependent channel activation and channel availability of Ca_v_1.3_L_ channels. The chord conductance values of Ca_v_1.3_L_ channels without (□) or with Snapin2 (■) were normalized to the maximal chord conductance and superimposed. A similar two-step pulse protocol was applied to evaluate channel availability of Ca_v_1.3_L_ channels without (□) or with Snapin2 (■). Normalized data for channel availability were plotted against pre-pulse potentials, and the smooth curves were from fitting the data to the Boltzmann equation (n = 20 for activation, n = 17 for channel availability).

We also examined whether Snapin2 can modulate Ca_v_1.3_L_ channel activity in *Xenopus* oocytes, an expression system quite different from HEK-293 cells. Ca_v_1.3_L_/Ca_v_β_3_ were co-expressed with or without Snapin2 by injection of their cRNAs in equal molar ratio. On the 4th day after cRNA injection, expression of Ca_v_1.3_L_ channels in oocytes was detected as robust Ba^2+^ currents in response to the *I-V* voltage protocol (Figure 3A). The *I-V* relationships obtained by plotting average peak current amplitudes against test potentials showed that average peak current amplitudes recorded from oocytes co-expressing Snapin2 with Ca_v_1.3_L_/β_3_ were about 2-fold greater at −10 mV test potential than those from the control oocytes expressed with Ca_v_3.1_L_/β_3_ (−2414.5 ± 314.6 vs. −1271.1 ± 214.1 nA) (Figure 3B,C). Comparison of chord conductance displayed that Snapin2 similarly increased the maximal chord conductance of Ca_v_1.3_L_ channels (14.6 ± 2.6 vs. 28.1 ± 2.9 μS) (Figure 2D). Similar increments were detected at other test potentials, but no significant alterations in voltage-dependent gating properties were induced by co-expression of Snapin2 (Figure 3E and Table 1). Taken together, our findings that Snapin2 similarly up-regulated Ca_v_1.3_L_ channel activity in two different expression systems implies that, regardless of cell type, the interaction of Snapin2 with the CT_L_ of Ca_v_1.3_L_ can increase Ca_v_1.3_L_ channel activity. Consequently, the up-regulation of calcium influx through Ca_v_1.3_L_ channels by Snapin2 might affect related physiological events.

### 2.4. Lack of the Snapin2 Up-Regulation Effect on Ca_v_1.3_S_ Variant

**Figure 4:**
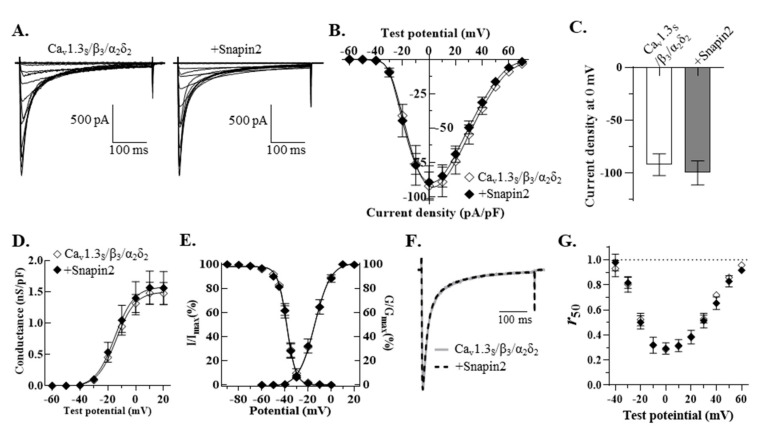
No regulatory effect of Snapin2 on Ca_v_1.3_S_. (**A**) Representative current traces through Ca_v_1.3_S_/β_3_/α_2_δ_2_ co-expressed without (left) or with Snapin2 (right). Ca_v_1.3_S_ current traces were evoked in response to the *I-V* protocol (described in Figure 2) in 10 mM Ca^2+^ solution. (**B**,**C**) *I-V* relationships of Ca_v_1.3_S_ channels without (◇) or with Snapin2 (◆). Average current density values (pA/pF) were plotted against test potentials (n = 8). (**C**) The average peak current density values at 0 mV test potential are represented as bar graphs (−92.1 ± 10.2 vs. −89.3 ± 11.9 pA/pF; n = 8). (**D**) No effect of Snapin2 on the chord conductance of Ca_v_1.3_S_ channels. The chord conductance values were normalized to the cell capacitance and plotted against test potentials (1.5 ± 0.2 vs. 1.6 ± 0.3 nS/pF; n = 8). (**E**) Activation and channel availability curves of Ca_v_1.3_S_ channels co-expressed without or with Snapin2. Activation and channel availability curves of Ca_v_1.3_S_ channels were obtained by the same methods in Figure 2 legend (n = 8). (**F**) Snapin2 effect on the CDI of Ca_v_1.3_S_ channels. Ca_v_1.3_S_ Ca^2+^ current traces elicited at 0 mV test potential were superimposed, exhibiting no difference in CDI. (**G**) No alteration in the *r*_50_ value of Ca_v_1.3_S_. The *r*_50_ values of Ca_v_1.3_S_ channels (±Snapin2) were plotted against test potentials (n = 5). No differences in the *r*_350_ values were detected (data not shown).

**Figure 5:**
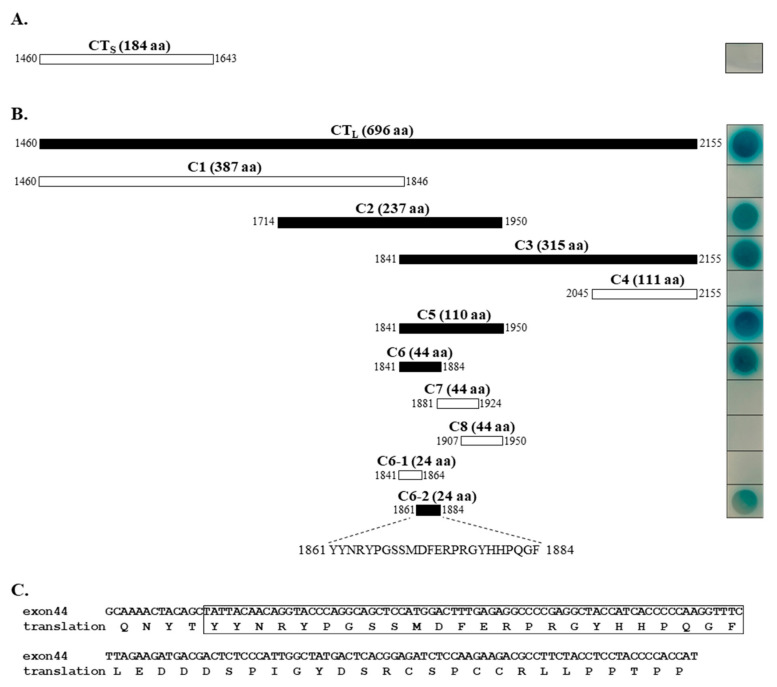
Identification of a Snapin2 interaction site in Ca_v_1.3 CT tails using Y2H assays. (**A**) No interaction of the CT_S_ of rat Ca_v_1.3_S_ with Snapin2, based on Y2H assays. The CTs of rat Ca_v_1.3_S_ are shown schematically as a white box with amino acid numbers and size. No blue colony was formed by Y2H assay (right). (**B**) Localization of a Snapin2 association site in the CT_L_ of Ca_v_1.3_L_. The CT_L_ of Ca_v_1.3_L_ is shown as a black box representing its association with Snapin2. The CT_L_ (1460–2155) was divided into C1 to C4. The overlapped region between C2 and C3 was designated as C5 (1841–1950), which was subsequently dissected into C6 to C8. C6 was finally divided into C6-1 and C6-2. C6-2 (YYNRYPGSSMDFERPRGYHHPQGF) was localized to interact with Snapin2. Blue colonies grown in a stringent condition (lacking Ade, His, Leu, and Trp) treated with X-gal were exhibited in the right side of the diagram. (**C**) Sequence information of C6-2 and exon44. The amino acid sequence of C6-2 marked as a box belongs to the deduced sequence of exon44.

We next investigated whether Snapin2 can regulate Ca_v_1.3_S_ variant containing a short CT. Co-expression of Snapin2 with Ca_v_1.3_S_/β_3_/α_2_δ_2_ did not show any alterations in the peak current density of Ca_v_1.3_S_ channels and other biophysical properties, including *I-V* relationship, CDI, and gating properties (Figure 4; Table 1). The differential regulatory effects by Snapin2 are likely to depend on the structural difference of CT tails. In order to find out in detail any differential regulation resulting from differences in the CT tails, we tested whether Snapin2 can interact with the short CT (designated as “CT_S_”) of Ca_v_1.3_S_ using Y2H assays. It was determined that the CT_S_ did not interact with Snapin2 (Figure 5A).

Subsequently we dissected the CT_L_ into multiple fragments and cloned them into bait vector to localize the Snapin2 interacting site in the CT_L_ using Y2H assays (Figure 5B). Systematic Y2H assays narrowed down a Snapin2 interaction site to be C6-2 comprised of 24 amino acids (YYNRYPGSSMDFERPRGYHHPQGF) (Figure 5B). Analysis of the amino acid sequence revealed that it pertained to the translated sequence of exon 44 (Figure 5C). These results strongly suggest that Snapin2 specifically interacts with long C-terminal Ca_v_1.3 variants containing exon 44 and explains why Ca_v_1.3_S_ variant deficient of exon 44 was not up-regulated by Snapin2.

### 2.5. The Snapin2 Up-Regulation of Ca_v_1.3_L_ Channel Activity Was Reproduced by Snapin1

A previous study using Y2H and GST pull-down assays reported that Snapin1, a longer variant than Snapin2, interacted with the CT_L_ of human Ca_v_1.3_L_ variant [30]. In contrast with our patch-clamping results showing that Snapin2 co-expression up-regulated Ca_v_1.3_L_ channel activity, Sun and his colleagues showed that Snapin1 co-expression strongly down-regulated Ca_v_1.3 channel activity in HEK-293 cells and HL-1 cells.

**Figure 6:**
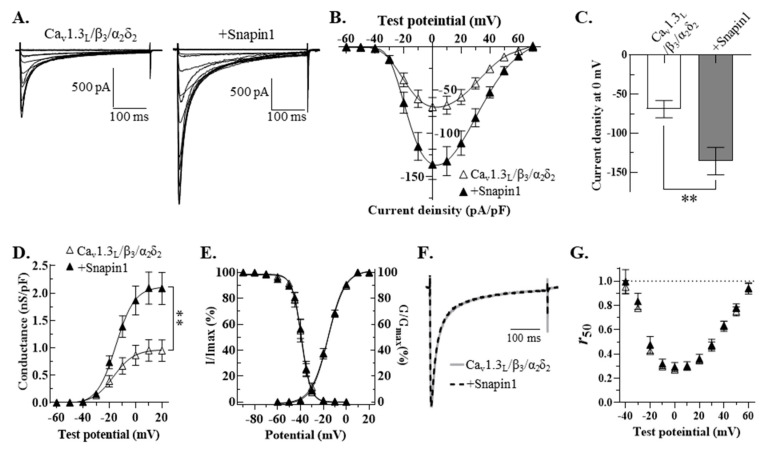
Up-regulation effect of Snapin1 variant on Ca_v_1.3_L_ channels. (**A**) Ca_v_1.3_L_/β_3_/α_2_δ_2_ without (left) or with (right) Snapin1 were co-expressed in HEK-293 cells. The same voltage protocols and analysis described in Figure 2 were used to characterize the biophysical properties of Ca_v_1.3_L_ channels. (**B**) The *I-V* relationships of Ca_v_1.3_L_/β_3_/α_2_δ_2_ co-expressed with (▲) or without (∆) Snapin1 (n = 8). (**C**) Snapin1 effect on the maximal current density of Ca_v_1.3_L_. The maximal current density values of Ca_v_1.3_L_ without or with Snapin1 are −69.2 ± 11.0 pA/pF and −135.7 ± 17.6 pA/pF (n = 10; Student’s unpaired *t*-test, *p <* 0.01, **). (**D**) The chord conductance of Ca_v_1.3_L_ channels without (∆) or with (▲) Snapin1. The maximal normalized conductance values of Ca_v_1.3_L_ without or with Snapin1 are 0.95 ± 0.20 and 2.09 ± 0.29 nS/pF (n = 10; Student’s unpaired *t*-test, *p <* 0.01, **). (**E**) Channel availability and activation curves of Ca_v_1.3_L_ channels without (∆) or with (▲) Snapin1 (n = 10). (**F**,**G**) No alteration in the CDI of Ca_v_1.3_L_ by Snapin1. Ca^2+^ current traces through Ca_v_1.3_L_ channels without or with Snapin1 evoked at 0 mV test potential were superimposed. The *r*_50_ values (**G**) of Ca_v_1.3_L_ channels in the presence or absence of Snapin1 at various test potentials did not change the CDI of Ca_v_1.3_L_ channels (n = 7).

Because these contradictory results might arise from different regulation by the two Snapin variants co-expressed, we cloned rat Snapin1 cDNA (by RT-PCR from rat brain total RNA; refer to the method section) to examine the regulatory effects of Snapin1 on Ca_v_1.3_L_ channels. In HEK-293 cells Snapin1 and Ca_v_1.3_L_/β_3_/α_2_δ_2_ were co-expressed by transfection of their constructs. In response to the *I-V* protocol, Ca_v_1.3_L_ channel currents were evoked from HEK cells under similar experimental conditions and showed that the Ca_v_1.3_L_ Ca^2+^ current density (−69.2 ± 11.0 vs. −135.7 ± 17.6 pA/pF) and maximal chord conductance (1.0 ± 0.2 vs. 2.1 ± 0.3 nS/pF) were approximately 2-fold up-regulated by Snapin1 (Figure 6B–D). In contrast, the CDI and voltage-dependent gating properties were little changed (Figure 6E–G). The results show that the up-regulation of Ca_v_1.3_L_ channel activity by Snapin1 is quite similar to that by Snapin2. We also tested whether Ca_v_1.3_S_ channel can be affected by co-expression of Snapin1. It turned out that Snapin1 did not alter the current density and other biophysical properties of Ca_v_1.3_S_ channel (Supplementary Figure S1). This implies that Snapin1 is likely not to interact with Ca_v_1.3_S_. These findings strongly suggest that both of the Snapin isoforms can facilitate the Ca_v_1.3_L_ channel activity in a similar manner via an association with the CT of Ca_v_1.3_L_.

### 2.6. No Effect of Snapin on Membrane and Total Expression of Ca_v_1.3_L_ Channels

**Figure 7:**
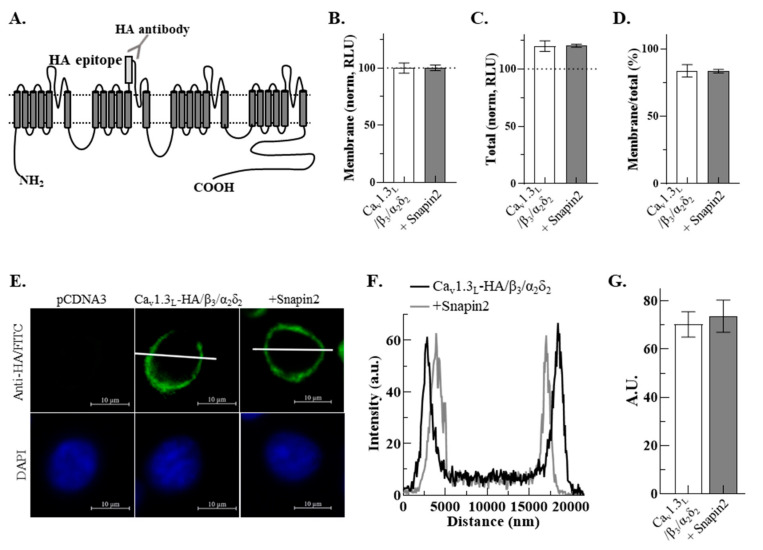
The surface and total expression of Ca_v_1.3_L_ channels was not changed by Snapin2. (**A**) Schematic diagram of Ca_v_1.3_L_-HA. The HA epitope was tagged at the extracellular S5-pore loop of Ca_v_1.3_L_ domain II (refer to the method section). (**B**–**D**) Luminometric quantification of expression levels of Ca_v_1.3_L_-HA without or with Snapin2. (**B**) Surface expression measured in non-permeabilized HEK-293 cells expressing Ca_v_1.3_L_-HA/β_3_/α_2_δ_2_ without or with Snapin2. The luminescence intensities (arbitrary unit) were 3.75 × 10^7^ ± 0.25 × 10^7^ and 3.86 × 10^7^ ± 0.20 × 10^7^ (n = 8). Their normalized values (relative luminescence unit, RLU) are represented as bar graphs. (**C**) Total expression measured in permeabilized HEK-293 cells expressing Ca_v_1.3_L_-HA/β_3_/α_2_δ_2_ without or with Snapin2. The luminescence values of total expression are 3.95 × 10^7^ ± 0.05 × 10^7^ and 4.35 × 10^7^ ± 0.20 × 10^7^ (n = 8). RLU values were obtained by normalizing luminescence values to the non-permeabilized Ca_v_1.3_L_-HA/β_3_/α_2_δ_2_ signal. (**D**) Ratios of surface/total expression are represented as bar graphs. The ratios are not significantly different between with or without Snapin2. (**E**) Confocal microscopy images of surface expression of Ca_v_1.3_L_-HA without or with Snapin2. A set of HEK-293 cells transfected with Ca_v_1.3_L_-HA/β_3_/α_2_δ_2_ without or with Snapin2 was stained with anti-HA antibodies and then FITC conjugated secondary antibodies. The middle and right panels show representative fluorescence images for Ca_v_1.3_L_-HA with or without Snapin2 (n = 3). The negative control image was from cells transfected with pCDNA3 vector (left panel). DAPI stained images of corresponding cells are shown in the lower panels. (**F**) Line-scanning analyses of Ca_v_1.3_L_-HA expression. Representative line-scan profiles of cells expressing Ca_v_1.3_L_-HA without (black line) or with Snapin2 (gray line) were obtained by a software program provided by ZEISS. (**G**) The maximal intensity values (arbitrary unit, a.u.) across the cells are represented as bar graphs, showing no statistically significant difference (70.3 ± 5.3 and 73.7 ± 6.6 a.u.; n = 10).

We engineered to fuse hemagglutinin (HA) epitope at the extracellular S5-pore loop of domain II of Ca_v_1.3_L_ (Figure 7A; refer to the method section) to examine whether the membrane and total expression of Ca_v_1.3_L_ channels can be changed by Snapin2. After Ca_v_1.3_L_-HA/β_3_/α_2_δ_2_ were co-expressed with or without Snapin2 in HEK-293 cells, membrane Ca_v_1.3_L_-HA channels and total Ca_v_1.3_L_-HA were quantitated by measuring luminescence intensity of multiple groups of non-permeabilized and permeabilized HEK-293 cells, respectively. Analysis of data showed that both the membrane expression and the total expression of Ca_v_1.3_L_-HA channels in HEK-293 cells were little changed by co-expression of Snapin2 (Figure 7B,C). Consistently, the normalized ratio of membrane Ca_v_1.3_L_-HA to total Ca_v_1.3_L_-HA was not affected by co-expression of Snapin2 (Figure 7D), supporting that Snapin2 does not affect the membrane and total expression of Ca_v_1.3_L_ channels.

To directly observe immunofluorescence images for their surface expression, we also performed immunocytochemistry staining experiments with HA antibodies and FITC conjugated secondary antibodies. Confocal microscopy exhibited little detection of fluorescence images taken from the control cells transfected with empty vector (left panel of Figure 7E). In contrast, there was strong detection of fluorescence images from the cells expressing Ca_v_1.3_L_-HA /β_3_/α_2_δ_2_ with or without Snapin2 (middle and right panels of Figure 7E), thereby indicating great surface expression of Ca_v_1.3_L_-HA channels. Notably, the membrane expression of Ca_v_1.3_L_-HA channels with or without co-expression of Snapin2 appeared to be comparable. When the fluorescence intensity of membrane Ca_v_1.3_L_ expression was quantitated using line-scanning analysis, the whole line-scan profiles for the membrane Ca_v_1.3_L_ expression with or without co-expression of Snapin2 were almost overlapped (Figure 7F). Analysis of data showed that the maximal fluorescence intensity in the plasma membrane was also comparable with or without Sapin2 (70.3 ± 5.3 vs. 73.7 ± 6.6 a.u.; Figure 7G). Taken together, these immunostaining results are in good agreement with the results from the luminometry experiments, strongly suggesting that co-expression of Snapin does not affect the surface expression and total expression of Ca_v_1.3_L_ channels.

### 2.7. Snapin Up-Regulation of Ca_v_1.3_L_ Activity Comes from Enhanced Opening Probability

**Figure 8:**
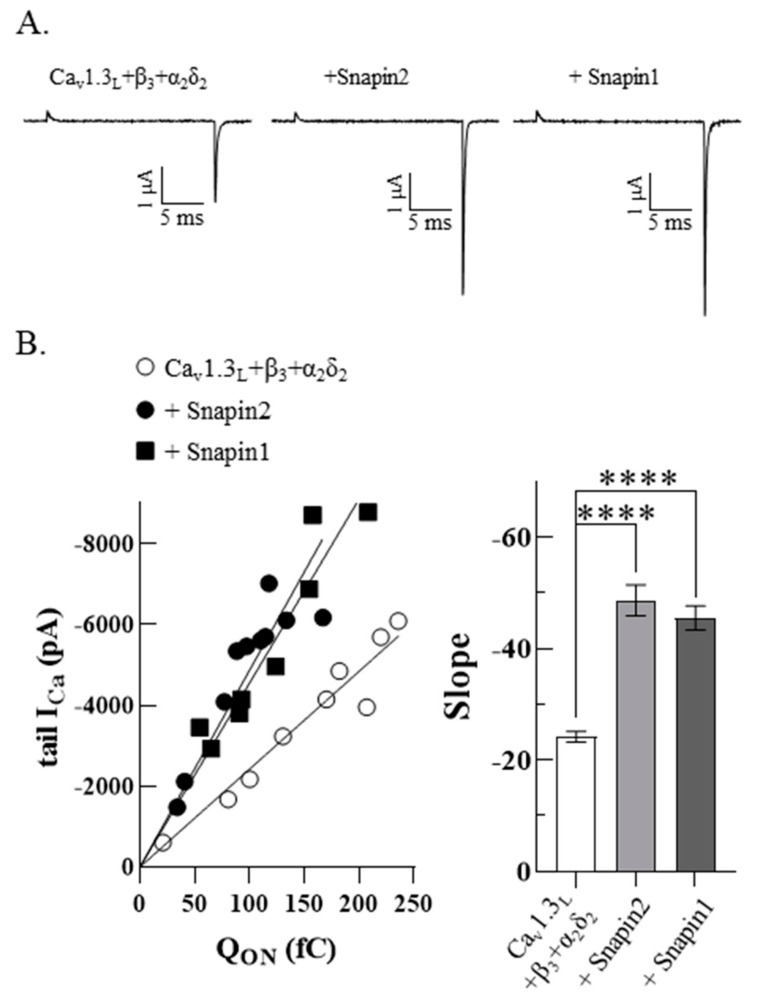
Increment of opening probability of Ca_v_1.3_L_ channels by Snapin. (**A**) Representative gating current and tail current of Ca_v_1.3_L_/β_3_/α_2_δ_2_ co-expressed without or with Snapin 1 or 2. ON-gating currents were measured upon depolarizing to the virtual reversal potential from a holding potential of −80 mV and tail currents were measured upon repolarizing to −80 mV holding potential from the same cells. (**B**) Correlation of ON-gating currents and tail currents. Q_ON_ (fC) values were plotted against tail current amplitudes (left panel, n = 8–10). The slope values obtained from linear regression of data are −24.2 ± 1.0 for Ca_v_1.3_L_/β_3_/α_2_δ_2_, −48.7 ± 2.7 for Ca_v_1.3_L_/β_3_/α_2_δ_2_ plus Snapin2, and −45.5 ± 2.2 for Ca_v_1.3_L_/β_3_/α_2_δ_2_ plus Snapin1 and are represented as bar graphs. Statistical significances were tested with one-way ANOVA with Tukey’s multiple comparison (*p <* 0.0001, ****).

Whole-cell current (*I*) can be described by the multiplication of the number of channels (*n*), their opening probability (*P*_o_), and the channel’s unitary conductance (*i*). Based on this relationship (*I = nP_o_i*), the increase of Ca_v_1.3_L_ activity (*I*) induced by co-expression of Snapin2, could be generated by changes of one or more parameters. To explore the underlying mechanism for the Snapin2 up-regulation of Ca_v_1.3_L_ activity, we attempted to measure ON-gating currents at the virtual reversal potential for the Ca^2+^ currents and ionic tail currents from the same cells where ON-gating currents (Q_ON_) are proportional to the number of channels in the plasma membrane and ionic tail currents (*I*_tail_) upon repolarization represent the whole channel activity (*I*) [32]. In general, we observed that Snapin co-expression produced larger tail currents against comparable ON-gating currents (Figure 8A). The Q_ON_ values obtained from integrating the areas under the ON-gating currents were plotted against tail current amplitude values from the same cells. Regression analysis of data yielded slopes, which represent approximate estimates of opening probability (*P_o_*) (Figure 8B). It turned out that the slopes were about 2-fold increased by co-expression of Snapin1 or Snapin2, indicating that the *P_o_* of Ca_v_1.3_L_ channels was significantly enhanced by Snapin1 or 2 (*p* < 0.0001, ****; Figure 8A,B). These results provide strong evidence supporting the underlying mechanism for the Snapin up-regulation effect on Ca_v_1.3_L_ activity comes from an increase in the opening probability (*P_o_*) of Ca_v_1.3_L_ channels.

## 3. Discussion

We here isolated rat Snapin2 as a molecular partner interacting with the CT_L_ of rat Ca_v_1.3_L_ by utilizing Y2H screening. In the two different expression systems, both Snapin1 and Snapin2 enhanced Ca_v_1.3_L_ channel activity, without altering other biophysical properties including voltage-dependent gating properties, current kinetics, and CDI. The unique interaction site of Snapin in CT_L_ was localized to a 24-amino-acid sequence, which belongs to exon 44, rendering the Ca_v_1.3_L_ channels to be specifically up-regulated by Snapin. Furthermore, combined studies of luminometry and measurement of ON-gating and tail currents revealed that Snapin enhances the opening probability of the Ca_v_1.3_L_ channel without changing the level of surface expression.

Our result showing that Snapin2 interacts with the CT_L_ of rat Ca_v_1.3_L_ is consistent with a previous report in which rat Snapin1, a longer variant than Snapin2, interacted with the CT_L_ of human Ca_v_1.3_L_ variant [30]. However, conflicting results were found in functional analysis of Snapin variants in expression systems. In our study, Snapin2 clearly up-regulated Ca_v_1.3_L_ channel activity, whereas in the previous study, Snapin1 strongly down-regulated Ca_v_1.3 channel activity. In contrast with the down-regulation result in the previous study, our subsequent study showed that the Snapin1 variant also up-regulated Ca_v_1.3_L_ channel activity.

To address possible causes for the conflicting results, we compared and analyzed patch-clamping data from the previous study with typical Ca_v_1.3 channel data in previous literature and from our experiments. First, we note the expression level and biophysical properties of Ca_v_1.3 channels. In the previous study, the currents estimated from HEK-293 cells stably expressing Ca_v_1.3_L_/β_2a_/α_2_δ were shown to be very small in average maximal current density (~3 pA/pF) and to be slowly activated upon +10 mV (or 0 mV) test potential [30]. The properties of Ca_v_1.3 current with tiny amplitude and slowly activating kinetics have not been shown in the other previous studies [11,33] or/and in our experiments. To use model cell lines to investigate voltage-activated ion channels, it is common knowledge that adequate expression levels and typical electrophysiological properties of certain channels would be primary prerequisites. To consider weak points, we also speculate that heterogeneity or impairment in the Ca_v_1.3 channel expression might lead to the low expression and abnormal property of the Ca_v_1.3 channel in the cell line used in the previous study. In addition, even if there is no causal relationship, only by considering the changes in the Ca_v_1.3 channel properties themselves, this implies that the experimental conditions were not appropriate. Second, we considered the suitability of HL-1 cells for studying Ca_v_1.3 channels. HL-1 cells, an atrial myocyte cell line, have been used for functional studies of T-type and L-type channels as well as atrial myocyte properties [34]. Among the L-type channels, the protein expression of the Ca_v_1.2 subunit was found to be greater than that of the Ca_v_1.3 subunit in the plasma membrane of HL-1 cells [34]. Thus, it can be inferred that the very small current (~1 pA/pF) was likely to be mainly permeated through the Ca_v_1.2 rather than the Ca_v_1.3 channel. This suggests that the HL-1 cell line is not suitable for Ca_v_1.3 regulation studies and raises the possibility that in the previous study, Snapin reduced Ca_v_1.2 channel activity via its interaction with the Ca_v_1.2 subunit. Thus, we tested the possibility of whether co-expression of Snapin1 with Ca_v_1.2/β_3_ can increase Ca_v_1.2 channel activity in *Xenopus* oocytes. The experimental results from the oocytes showed that Snapin co-expression did not cause any statistically significant changes in the current amplitude and voltage-dependent gating properties of Ca_v_1.2 channels (Supplementary Figure S2). No regulation of Ca_v_1.2 current by Snapin1 does not support the tentative hypothesis and is contradictory to the previous report addressing that Snapin1 down-regulated L-type channel activity by ~50% in HL-1 cells [30]. Even assuming that Snapin1 completely down-regulated Ca_v_1.3 activity, total L-type channel current in HL-1 cells should have been mildly down-regulated by Snapin1 because Ca_v_1.2 current in HL-1 cells would remain unaffected by Snapin1 (Supplementary Figure S2). Thus, we speculate that the contradictory effects might arise from Snapin-mediated nonspecific pathways, influencing channel activity of both L-type Ca_v_1.2 and Ca_v_1.3 isoforms.

We conducted further investigations to uncover the underlying mechanism for the Snapin up-regulation effect on Ca_v_1.3_L_ using luminometry and immuno-cytochemistry to quantitate the surface and total expression of Ca_v_1.3_L_ and electrophysiological measurements of ON-gating currents and tail currents at the reversal potential to estimate channel-opening probability (*P_o_*) [32]. Greater tail currents in the co-expression of Snapin were observed from comparable ON-gating currents. Analyzed from the relationships of ON-gating currents versus tail currents from the same cells, co-expression of Snapin induced about a 2-fold increase in the opening probability of Ca_v_1.3_L_ channels, implying that the Snapin interaction exerted an effect that coupled charge movement to channel opening in a more efficient way. Combined with the results of no changes in the membrane and total expression, these results strongly suggest that Snapin can up-regulate Ca_v_1.3_L_ channel activity through its specific association with long C-terminal Ca_v_1.3_L_ variants, coupled with an increase of opening probability.

The Snapin interaction with SNAP25, one of the protein components composing SNARE complex, has been functionally relevant to facilitating transmitter release by increasing the number of priming and fusion synaptic vesicles [21,22,26,35] and inducing a fast exocytosis of large dense-core vesicles in chromaffin cells and insulin secretion of pancreatic beta cells [20,36,37]. Besides those roles of Snapin, we here report a novel role of Snapin, that is, an augmentation of Ca^2+^ entry through Ca_v_1.3_L_ channels. Although it could be speculated simply that this role of Snapin may impact synaptic transmission and hormone secretion, it should be considered that the main Ca^2+^ channel types for synaptic release are N- and P/Q-type channels but not L-type channels [1]. Thus, we carefully suggest that this novel role of Snapin may cause a slight increase in the basal calcium levels of neurons. This can form favorable conditions for neuronal Ca^2+^ signaling pathways, which could plausibly lead to facilitation of basal synaptic transmission, modulation of gene expression, and neuronal development [1,8,38,39]. Nevertheless, in certain neuronal cells, such as cochlear inner hair cells and retinal bipolar cells, where Ca_v_1.3 channels are expressed [2,40], it is possible that the newly discovered role of Snapin directly contributes to facilitating synaptic transmission. Additionally, in adrenal chromaffin cells, where Ca_v_1.3_L_ channels are expressed and involved in generation of pacemaking activity and catecholamine secretion [12], that the new role of Snapin may contribute to accelerating pacemaking activity and facilitating catecholamine secretion.

We conclude that Snapin2 specifically associates with long C-terminal Ca_v_1.3 variants and up-regulates the channel activity. The up-regulation effect of Snapin on Ca_v_1.3_L_ arises from the increased opening probability without changes in the membrane and total expression. The enhanced Ca_v_1.3 activity in the presence of Snapin may be a novel mechanism underlying the facilitation effects of Snapin on synaptic transmission and hormone secretion.

## 4. Materials and Methods

### 4.1. Chemicals

Most of the chemicals were purchased from Sigma-Aldrich (St. Louis, MO, USA) and Thermo Fisher Scientific (Waltham, MA, USA). Minimal nutrients used for yeast-two-hybrid (Y2H) screenings were from Clontech (Mountain View, CA, USA).

### 4.2. Yeast Two-Hybrid (Y2H) Screening

The long carboxyl terminus (CT_L_) of rat Ca_v_1.3_L_ variant (GenBank accession no. NM_001389225; Addgene plasmid no. 49332; Xu and Lipscomb, 2001) was amplified by PCR (polymerase chain reaction) and then cloned into bait pGBKT7 vector using NdeI and BamHI sites. CT_L_–pGBKT7 construct and rat brain cDNA library in pACT2 vector (Clontech) were co-transformed to AH109 competent cells to isolate novel proteins interacting with CT_L_ using Matchmaker GAL4 yeast two-hybrid system (Clontech). A positive colony was selected on a high-stringent SD medium deficient of Leu, Trp, His, and Ade, in the presence of X-α-Gal. The vector insert in the positive clone was identified as including the full-length cDNA of rat Snapin2 (GenBank accession no. NM_001025648). The Snapin2 cDNA was subcloned into pcDNA3 and pGEM-HEA vectors for co-expression studies in HEK293-cells and *Xenopus* oocytes, respectively.

The N-terminus (NT), I-II loop, II-III loop, and III-IV loop of Ca_v_1.3_L_ and the short CT (CT_S_) of rat Ca_v_1.3_S_ variant (Addgene plasmid number 49,333; GenBank accession no. AF370009; [10]) were also amplified by PCR and individually inserted into pGBKT7 bait vector. Their interaction with rat Snapin2 was tested by Y2H assays.

### 4.3. Cloning of Rat Brain Snapin1

Rat brain cDNA was reverse-transcribed from rat brain total RNA (Clontech) using One-step RT-PCR kit (Qiagen, Hilden, Germany) according to the manufacturer’s protocol. PCR was employed to amplify rat Snapin1 cDNA using forward and reverse primers (5′-GCGTCAGGGCAGTTCGC-3′ and 5′-TTCTGTCTACCAAGACAAAGCTGT-3′). The PCR products were cloned into pCR2.1-TOPO vector (Invitrogen). One of the PCR products in pCR2.1-TOPO vector was analyzed by sequencing and then subcloned into pCDNA 3.1 vector using EcoRI and XbaI sites. 

### 4.4. Co-Expression of Snapin2 or Snapin1 with Ca_v_1.3 Variants in HEK-293 Cells

HEK-293 cells were cultured at 37 °C, 5% CO_2_ in DMEM media containing 10% FBS, 1% penicillin, and streptomycin. Rat Snapin2- or Snapin1-pcDNA3 (or pcDNA3 null vector, control) was co-transfected into HEK-293 cells with cDNA mixture of rat Ca_v_1.3_L_ (or Ca_v_1.3_S_), rat Ca_v_β_3_ (GenBank accession no. M88751), and human Ca_v_α_2_δ_2_ (GenBank accession no. NM_006030) at equal molar ratio, together with 1/100 of eGFP-pcDNA3 construct using Effectene reagent (Qiagen) according to the provided protocol. Thirty-six hours after transfection, cells were dissociated from culture dishes with 0.25% trypsin-EDTA and plated on poly-L-lysine treated coverslips. Patch clamp recordings were performed between 48~72 h after transfection.

### 4.5. Co-Expression of Snapin2 with Ca_v_1.3_L_ in Xenopus Oocytes

Rat Ca_v_1.3_L_-pCDNA3 construct was linearized by *Avr*II at the 3′ end. Rat β_3_ and Snapin2 constructs were linearized by *Sac*II and *Afl*II, respectively. The linearized cDNAs were used as substrates for capped transcript synthesis using mMESSAGE mMACHINE T7 transcription kit (Ambion, Austin, TX USA) according to the manufacturer’s instruction. 

*Xenopus laevis* frogs were purchased from Hallym University (Chuncheon, Korea). As described previously [41], several ovary lobes were surgically isolated from female frogs, which were anesthetized. Follicles were subsequently eliminated from oocytes by digestion with collagenase (Gibco, 12 mg/mL) in a Ca^2+^-free OR2 solution (in mM: 82.5 NaCl, 2.5 KCl, 1 MgCl_2_, 5 HEPES; pH 7.6 with NaOH). Prior to cRNA injection, oocytes were recovered for several hours in a standard oocyte solution (in mM: 100 NaCl, 2 KCl, 1.8 CaCl_2_, 1 MgCl_2_, 5 HEPES, 2.5 pyruvic acid, and 50 mg/mL gentamicin; pH 7.6 with NaOH). 

Snapin2 cRNA (or water) was co-injected with cRNA mixture of Ca_v_1.3_L_ and Ca_v_β_3_ at equal molar ratio using a Drummond Nanoject injector (Parkway, PA, USA). Current traces from *Xenopus* oocytes were recorded from the 4th day after injection.

### 4.6. Electrophysiological Recordings in HEK-293 Cells

Whole-cell currents were measured with an Axopatch 200B patch clamp amplifier (Molecular Devices, Foster City, CA, USA) connected to a computer via a Digidata 1322A converter (Molecular Devices). The recording system and voltage-protocols were controlled using pCLAMP 9.2 software. Patch pipettes were pulled from borosilicate capillary tubing (World Precision Instruments, Sarasota, FL, USA) using PC-10 pipette puller (Narishige, Tokyo, Japan). When pipettes were filled with internal solution (in mM: 130 CsCl, 2 CaCl_2_, 10 HEPES, 10 EGTA, 5 MgATP; pH 7.3 with CsOH), the input resistance was 1.8–2.5 MΩ. The 10 mM CaCl_2_ external solution was used for recordings (in mM: 140 TEACl, 2.5 CsCl, 10 CaCl_2_, 1 MgCl_2_, 10 HEPES; pH 7.3 with TEAOH).

Current-voltage (*I-V*) relationship was obtained by 400-ms depolarizing step pulses from −60 mV to +70 mV by increments of 10 mV from a holding potential of −80 mV. Ca_v_1.3 currents were filtered at 2 kHz and sampled at 10 kHz. Ca^2+^-dependent inactivation (CDI) was quantified as the fraction (*r*_50_) of peak amplitude remaining after 50 ms plotted against various test potentials. Chord conductance values were calculated by the equation *G* = *I*/(*V*–*V*_rev_), where *I* is the current amplitude at a test potential *V*, and *V*_rev_ is the reversal potential. Channel availability was obtained by 10-s pre-pulses from −90 mV to −10 mV, followed by 0 mV test pulse from a holding potential of −80 mV.

Activation data were fitted by the Boltzmann equation: *G =* 1/[1 + exp{(*V_50,act_* − *V)/S_act_*}], where *V_50,act_* is the half-maximal activation potential, and *S_act_* is the slope conductance. Channel availability curves were fitted by the Boltzmann equation: *I*/*I_max_* = 1/[{1 + exp{(*V_50,inact_* − *V*)/*k*}], where *I*_max_ is maximal current amplitude, *V*_50,inact_ is the half-inactivation potential, and *k* is the slope factor. 

To estimate channel opening probability [32], ON-gating currents were measured by application of a 20-ms step pulse to the virtual reversal potential (+70~75 mV) and, from the same cells ionic tail currents (*I*_tail_), were measured upon repolarization to a holding potential of −80 mV. The capacitive linear properties were subtracted using −P/6 protocol. Data sampling for ON-gating currents was filtered at 10 KHz and sampled at 50 KHz. ON-gating currents at the reversal potential were represented as maximal charge movement values (Q_ON_), which were obtained from integrating the areas under ON-gating currents. The plots data of Q_ON_ values versus tail current amplitudes corresponding to maximal conductance were regressed to yield slopes, which are approximate estimates of opening probability (*P_o_*).

### 4.7. Electrophysiological Recordings in Xenopus Oocytes

A two-electrode voltage clamp method was applied to record Ca_v_1.3_L_ current traces from *Xenopus* oocytes in 10 mM Ba^2+^ recording solution (in mM: 90 NaOH, 1 KOH, 10 Ba(OH)_2_, 5 HEPES; pH 7.4 with CH_3_SO_3_H). Electrodes pulled from borosilicate capillary glass tubing (Warner Instrument, Holliston, MA) were filled with 3M KCl, and their resistance was 0.8~1.2 KΩ. Prior to current recordings, oocytes were injected with 40 mM BAPTA solution in a volume of 50 nL to eliminate endogenous Ca^2+^ activated Cl^−^ current. Ca_v_1.3 Ba^2+^ currents were filtered at 1 kHz and sampled at 5 kHz. Data were analyzed with Clampfit 10.7 software and Prism software (GraphPad, San Diego, CA, USA). 

### 4.8. Construction and Luminometric Measurement of Ca_v_1.3_L_-HA

The hemagglutinin (HA) epitope was inserted between amino acids Q693 and T694 at the extracellular S5-pore loop of domain II by overlap extension PCR [42]. Snapin2-pCDNA3 (or empty pCDNA3) was co-transfected with Ca_v_1.3_L_-HA/β_3_/α_2_δ_2_ constructs into HEK-293 cells using Effectene (Qiagen). Forty-eight hours after transfection, cells were washed with PBS and fixed in 4% paraformaldehyde for 5 min. A set of cell samples was permeabilized with 0.1% Triton X-100 for 5 min, and the other set was non-permeabilized without Triton treatment. After being washed three times with PBS, cells were incubated for 30 min in 2% bovine serum albumin solution for blocking and incubated in polyclonal rabbit anti-HA antibody for 1 h (1:200 dilution; 71–5500, Thermo Fisher Scientific). After washings, cells were incubated with secondary goat anti-rabbit antibody conjugated with HRP (1:500 dilution; 170–6515, Bio-Rad) for 30 min and rinsed with PBS. After addition of SuperSignal ELISA femto maximum sensitivity substrates (Pierce, Rockford, IL, USA), the luminescence intensities of cells expressing Ca_v_1.3_L_-HA/β_3_/α_2_δ_2_ proteins were measured using EnSpire Multimode plate reader (PerkinElmer, Wellesley, MA, USA). The measured luminescence intensities were normalized with the amounts of protein in the individual wells determined by BCA assay.

### 4.9. Immunocytochemistry Analysis

Snapin2-pCDNA3 (or empty pCDNA3) was co-transfected with Ca_v_1.3_L_-HA/β_3_/α_2_δ_2_ constructs into HEK-293 cells. After 48 h, cells were washed with PBS and fixed with 4% paraformaldehyde for 5 min. After washing, two sets of cells were blocked with 2% bovine serum albumin (BSA) for 30 min, followed by overnight incubation polyclonal rabbit anti-HA antibody (1:200 dilution; 71–5500, Thermo Fisher Scientific) with 0.1% BSA at 4 °C. On the next day, cells were washed with PBS and incubated with FITC-conjugated goat anti-rabbit IgG antibodies (1:500 dilution; sc-2012, Santa Cruz, CA, USA) for 1 h. Cells were washed with PBS and covered with slides. Fluorescence images were taken using a confocal microscope (ZEISS LMS 880, Oberkochen, Germany). Line-scan analysis of the cell fluorescence images was obtained using ZEISS ZEN software and exhibited as relative fluorescence intensity (arbitrary unit, a.u.).

### 4.10. Statistical Analysis

All data values are presented as mean ± S.E.M. Statistical significances were evaluated using Student’s unpaired *t*-test or one-way ANOVA combined with Tukey’s multiple comparison tests, with *p <* 0.05 (*), *p <* 0.01 (**), *p <* 0.001 (***), and *p <* 0.0001 (****) as levels of significance.

## Figures and Tables

**Table 1:** Summary of biophysical properties of Ca_v_1.3 channels co-expressed with Snapin.

	V_50, act_ (mV)	Slope (*k*)	V_50,inact_ (mV)	Slope (*k*)	n	Maximal Current Density (pA/pF)	System
Ca_v_1.3_L_/β_3_/α_2_δ_2_	−16.0 ± 0.3	7.3 ± 0.3	−39.2 ± 0.3	−4.1 ± 0.3	18~21	−63.8 ± 6.4	HEK-293 cell
+Snapin 2	−16.2 ± 0.5	7.1 ± 0.4	−38.9 ± 0.5	−4.6 ± 0.5	12~15	−124.8 ± 15.9 (**)	
+Snapin 1	−15.9 ± 0.3	7.0 ± 0.3	−39.0 ± 0.4	−4.0 ± 0.4	10	−135.7 ± 21.2 (**)	
Ca_v_1.3_S_/β_3_/α_2_δ_2_	−14.2 ± 0.3	6.9 ± 0.2	−38.3 ± 0.3	−3.9 ± 0.3	8	−92.1 ± 10.2	
+Snapin 2	−14.3 ± 0.8	7.0 ± 0.7	−38.4 ± 0.4	−3.9 ± 0.3	8	−89.3 ± 11.9	
Ca_v_1.3_L_/β_3_	−18.4 ± 0.8	9.8 ± 0.8	−47.8 ± 1.5	−5.8 ± 1.3	17~20	−1271.1 ± 164.8 nA	*Xenopus* oocyte
+Snapin 2	−19.5 ± 1.0	9.5 ± 0.9	−47.2 ± 1.8	−5.7 ± 1.6	17~20	−2100.5 ± 209.7 nA (**)	

Data are presented as mean ± S.E.M. Significance levels were evaluated using Student’s *t*-test or one-way ANOVA combined with Turkey’s multiple comparison test; *p <* 0.01 (**).

## Data Availability

The data that support for this publication are available from the corresponding author under reasonable request.

## Supplementary Materials

The followings are available online at https://www.mdpi.com/article/10.3390/ijms222011268/s1, Figure S1: No regulatory effect of Snapin1 on Ca_v_1.3_S_. Figure S2: Snapin1 did not alter Ca_v_1.2 channel activity in *Xenopus* oocytes.
