# Supplementary material for: Snapin Specifically Up-Regulates Cav1.3 Ca2+ Channel Variant with a Long Carboxyl Terminus
Source: Int J Mol Sci. 2021 Oct 19;22(20):11268. doi: 10.3390/ijms222011268 (PMC8537452; doi:10.3390/ijms222011268)
Supplement: Supplementary file 1 [file ijms-22-11268-s001.zip › ijms-1404113-supplementary.pdf]

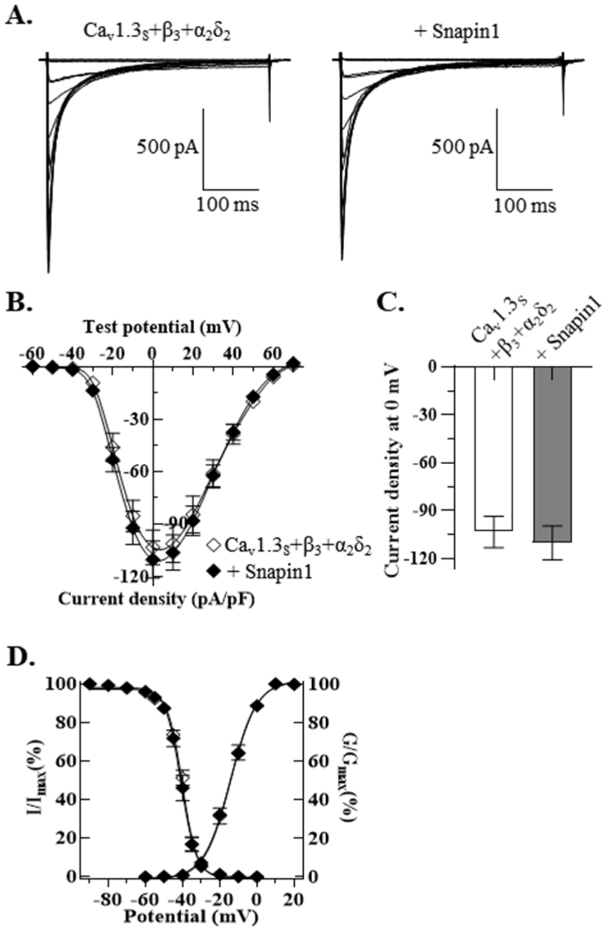

Supplementary Figure S1. No regulatory effect of Snapin1 on $Ca_v1.3_S$. (**A**) Representative Ca^{2+} current traces recorded from HEK-293 expressing $Ca_v1.3_S/\beta_3/\alpha_2\delta_2$ (left) or $Ca_v1.3_S/\beta_3/\alpha_2\delta_2$/Snapin1 (right). $Ca_v1.3_S$ current traces were evoked in response to the *I-V* protocol (described in Figure 2). (**B–C**) *I-V* relationships of $Ca_v1.3_S/\beta_3/\alpha_2\delta_2$ (◇) and $Ca_v1.3_S/\beta_3/\alpha_2\delta_2$/Snapin1 (◆). Current density values (pA/pF) were averaged and plotted against test potentials (n=6). (**C**) The average current density values at 0 mV test potential are represented as bar graphs (–103.4 ± 9.8 vs –110.4 ± 10.6 pA/pF; n = 6). (**D**) Activation and channel availability curves of $Ca_v1.3_S$ channels co-expressed without or with Snapin1. Activation and channel availability curves were obtained by the same methods in Figure 2 legend (n = 6).

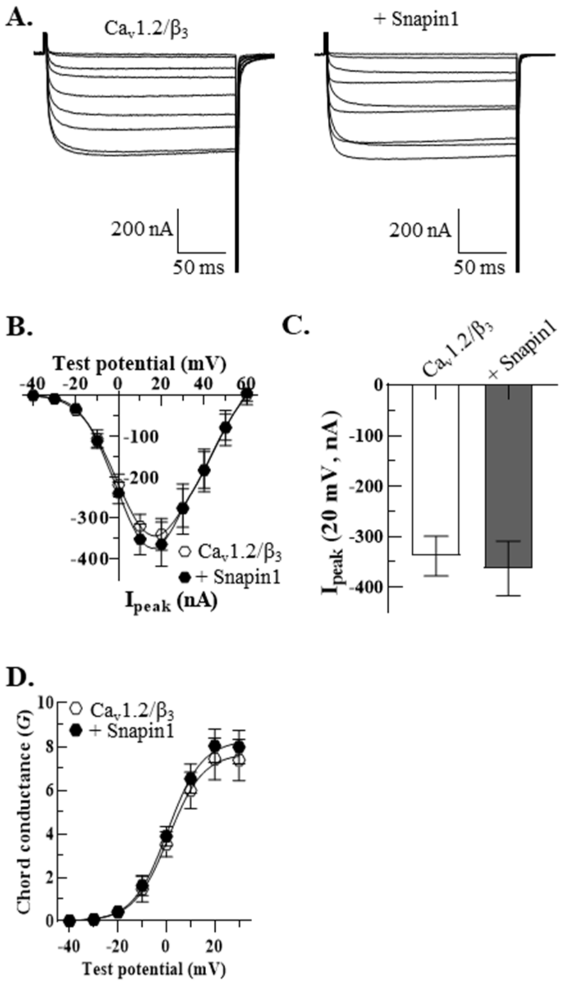

Supplementary Figure S2. Snapin1 did not alter $Ca_v1.2$ channel activity in *Xenopus* oocytes. (**A**) Representative current traces of $Ca_v1.2/\beta_3$ co-expressed without (left) or with Snapin1 (right) recorded in 10 mM Ba^{2+} solution. Current traces were evoked by an *I-V* protocol consisting of 200 ms depolarizing step pulses from –40 mV to +60 mV from a holding potential of –80 mV. (**B**–**C**) *I-V* relationships of $Ca_v1.2/\beta_3$ without or with Snapin1. (**B**) Peak current amplitude values of $Ca_v1.2/\beta_3$ and $Ca_v1.2/\beta_3$/Snapin1 were plotted against test potentials (n=9). (**C**) Maximal peak current amplitude of $Ca_v1.2/\beta_3$ and $Ca_v1.2/\beta_3$/Snapin1. Average peak current amplitude values at +20 mV test potential are represented as bar graphs (–338.7 ± 39.1 and –363.5 ± 54.1 nA). (**D**) Activation curves of $Ca_v1.2/\beta_3$ and $Ca_v1.2/\beta_3$/Snapin1. Activation curves were obtained by the same methods in Figure 2 legend (n = 6–8). The activation curves are almost overlapped.
